# Infantile Fructose-1,6-Bisphosphatase Deficiency Masquerading as Mitochondriopathy

**DOI:** 10.7759/cureus.67291

**Published:** 2024-08-20

**Authors:** Varshini Chandrasekhar, Pallavi Yelkur, Vidhyasagar K

**Affiliations:** 1 Paediatrics, Saveetha Medical College and Hospital, Saveetha Institute of Medical and Technical Sciences (SIMATS) Saveetha University, Chennai, IND

**Keywords:** mitochondriopathy, genetic testing, hypoglycemia, metabolic acidosis, fructose

## Abstract

Fructose-1,6-bisphosphatase 1 (FBP1) deficiency is a rare autosomal recessive disorder of gluconeogenesis. Affected children present with severe hypoglycemia and lactic acidosis in infancy. We report a case of a female child, aged one year and six months, born out of a third-degree consanguineous marriage, who initially presented with sudden-onset vomiting episodes and failure to thrive. Despite a clinical suspicion of mitochondrial disorder, biochemical investigations revealed elevated levels of alanine, glycine, lactic acid, pyruvic acid, 3-hydroxy isovaleric acid, fumaric acid, and 4-hydroxy phenylacetic acid. Clinical exome sequencing confirmed homozygous inheritance of a mutated *FBP1* gene, establishing the diagnosis of FBP1 deficiency. Differential diagnoses included mitochondrial disorders and transaldolase deficiency, but comprehensive genetic testing excluded these conditions. Management focused on dietary adjustments to avoid simple sugars and increase complex carbohydrates during illness. This case underscores the complexity of diagnosing rare metabolic disorders and highlights the pivotal role of genetic testing in accurate diagnosis and management.

## Introduction

Fructose-1,6-bisphosphatase 1 (FBP1) deficiency is an autosomal recessive disorder of fructose metabolism caused by mutations in the *FBP1* gene, which encodes the crucial enzyme FBP1. This enzyme is essential for regulating fructose metabolism [1]. The condition leads to severe, recurring episodes of hypoglycemia and lactic acidosis, typically triggered by infections, the introduction of complementary feeding, fever, fasting, reduced intake, vomiting, or high fructose consumption. Symptoms often manifest in early childhood and include hyperventilation, apneic spells, seizures, and coma. Nearly half of affected infants may present with hypoglycemia shortly after birth due to low glycogen reserves [2].

Individuals who experience recurring episodes of lactic acidosis and ketotic hypoglycemia and exhibit specific urinary organic acid profiles (such as peaks in glycerol and glycerol-3-phosphate) should be considered at high risk for FBP1 deficiency. Diagnosis is typically confirmed by identifying two pathogenic variants in the *FBP1* gene through genetic testing, which is preferred for its accessibility and reliability [3]. Alternatively, reduced FBP1 enzyme activity in the liver or mononuclear white blood cells can be measured. Key diagnostic features include hypoglycemia, high anion-gap metabolic acidosis, lactic acidemia, potential ketosis, and pseudo-hypertriglyceridemia due to elevated glycerol levels. Hyperuricemia and occasionally elevated free fatty acids may also be observed. These signs warrant further investigation as they could indicate FBP1 deficiency. If not diagnosed and treated promptly, affected children may progress from recurrent hypoglycemic episodes to seizures, coma, and potentially death [4,5].

Mutations in this gene were initially reported in 1970 [4], causing reduced or absent activity of fructose-1,6-diphosphatase in the liver. These genetic alterations prevent the conversion of fructose-1,6-diphosphate to fructose-6-phosphate, impacting gluconeogenesis [5]. While the condition is typically fatal in newborns, early diagnosis and rigorous management significantly improve the long-term outlook for this condition, underscoring the critical role of promptly confirming the molecular diagnosis [6,7].

## Case presentation

A female child aged one year and six months, the first child of a third-degree consanguineous marriage, presented with sudden-onset vomiting episodes. Her mother had an uncomplicated pregnancy and delivered her at full term via normal vaginal delivery, weighing 3.5 kg. The child experienced neonatal hyperbilirubinemia on day three of life, requiring two days of phototherapy in the neonatal intensive care unit before discharge from the hospital. Until 12 months of age, she had no complaints but then started experiencing recurrent, sudden-onset vomiting episodes and hypoglycemia, leading to dehydration and requiring hospitalization.

The child is developmentally normal and has received vaccinations according to the national immunization schedule. There was no significant family history of similar complaints among any family members on both the paternal and maternal sides. No prior workup was done on the parents, as they also did not have any similar complaints. Anthropometric measurements indicated failure to thrive based on weight, height, and weight-for-height ratio, all falling below the third standard deviation as per WHO growth charts. The general examination was found to be normal with no gross dysmorphic features; in addition, on the abdomen examination, there was no palpable hepatomegaly. When other systems were examined, they revealed findings within normal parameters.

A complete blood count, renal function test, and liver function test showed normal results. The C-reactive protein test was found to be negative, and sterile blood and urine cultures were obtained. Abdominal ultrasound indicated normal liver and spleen sizes without organ enlargement. Echocardiography revealed no cardiac abnormalities. Blood gas analysis revealed high anion-gap metabolic acidosis (Table 1), which was disproportionate to the vomiting episodes.

**Table 1:** Arterial blood gas (ABG) analysis

Components of Blood Gas	Blood Gas Values	Reference Values
pH	7.107	7.35-7.45
pCO_2_ (carbon dioxide)	30 mmHg	35-45 mmHg
pO_2_ (oxygen)	49.8 mmHg	80-100 mmHg
HCO_3_ (bicarbonate)	15.9 mmol/L	22-26 mmol/L
Anion gap	19.2	10-12
Lactate	7.1 mmol/L	0.5-1 mmol/L

Specialized biochemical tests conducted on the child's blood sample revealed markedly elevated levels of total carnitine, free carnitine, and acylcarnitine. This investigation was done using tandem mass spectrometry. Additionally, the ratio of free to acylcarnitine was found to be increased, as detailed in Table 2. In light of these findings, a thorough evaluation was initiated to explore potential underlying conditions, including carnitine deficiency, an inborn error of fatty acid beta-oxidation, and organic acidurias.

**Table 2:** Carnitine/acylcarnitine profile

Investigation	Actual Value (μmol/L)	Normal Range (μmol/L)
Total carnitine	114.95	20-87.7
Free carnitine	91.41	24.7-66.6
Acylcarnitine	23.54	4-28
Free/acyl ratio	3.88	>2.0

In addition to the above parameters analyzed, Table 3 reveals a derangement in amino acid and free fatty acid profile. The analysis shows elevated levels of alanine and glycine, with both exceeding their normal ranges. Non-esterified fatty acids (NEFA) are also higher than expected, although beta-hydroxybutyrate (BHB) is within the normal range. As a result, mitochondrial disorder was suspected due to initial findings of elevated carnitine and amino acid levels resulting in the initiation of supplementation with coenzyme Q and riboflavin.

**Table 3:** Amino acid and free fatty acid profile

Investigations	Actual Value (μmol/L)	Normal Range (μmol/L)
Alanine	2322.47	600.0
Glycine	1486.82	900.0
Non-esterified fatty acid	0.731	0.133-0.455
Beta-hydroxybutyrate	0.107	0.02-1.0
Non-esterified fatty acid/beta-hydroxybutyrate	6.83	0.5-5.0

Organic acid analysis was performed by using both liquid and gas chromatography methods to rule out organic acidurias, as detailed in Table 4. The results showed elevated levels of several organic acids, including lactic acid, pyruvic acid, 3-hydroxyisovaleric acid, fumaric acid, and 4-hydroxyphenylacetic acid, suggesting a possible co-existing organic acid defect in the child. Additionally, the levels of biotinidase enzyme, Gal-1-Put enzyme, and total galactose levels were identified by making use of mass spectrometry, and all were found to be within normal ranges.

**Table 4:** Organic acid analysis

Biochemical Parameter	Actual Value	Normal Range
Lactic acid	9.266 mmol/L	2 mmol/L
Pyruvic acid	11.234 mmol/L	0.08-0.16 mmol/L
3-hydroxyisovaleric acid	15.417 mmol/L	0.42-8.5 mmol/L
Urea	18.553 mg/dL	5-18 mg/dL
Fumaric acid	22.106 nmol/mg	0-16 nmol/mg
4-hydroxy phenylacetic acid	35.615 mmol/mol creatinine	0-29 mmol/mol creatinine

Table 5 details the positive findings from clinical exome sequencing, which was conducted to identify the biochemical defect contributing to the child's clinical symptoms and abnormal lab results. This analysis aimed to rule out inborn errors of metabolism and storage disorders. Using the IDT xGen Exome Research Panel v2.0 (Coralville, Iowa) for clinical whole-exome sequencing, a homozygous pathogenic variant in the *FBP1* gene (ENST00000415431.5) was identified. Specifically, the variant c.611_614del (p.Lys204ArgfsTer72) was found, with autosomal recessive inheritance. This mutation causes a frameshift, resulting in a premature stop codon at position 72 and the production of a truncated FBP1 enzyme. The truncated protein is typically nonfunctional or lacks the essential catalytic activity required for gluconeogenesis.

**Table 5:** Clinical exome sequencing showing FBP1 gene mutation OMIM: Online Mendelian Inheritance in Man

Gene (Transcript)	Location	Variant	Zygosity	Disease (OMIM)	Inheritance	Classification
FBP1 (-) (ENST00000415431.5)	Exon 6	c.611_614del (p.Lys204ArgfsTer72)	Homozygous	Fructose-1,6-bisphosphatase deficiency	Autosomal recessive	Pathogenic

Upon diagnosing FBP1 deficiency, a specific dietary plan was recommended for the patient. The plan emphasized frequent meals to prevent fasting and included incorporating more uncooked starches, such as rice powder, into her diet. She was advised to avoid simple sugars, such as table sugar, fruit juices, toffees, and chocolates, and instead focus on consuming complex carbohydrates and increasing vegetable intake. During follow-up visits, her growth and development were closely monitored, showing normal progress with normalized metabolic parameters. Her abdominal examination remained normal, with no indications of hepatomegaly or splenomegaly. The parents were instructed to maintain regular follow-ups and adhere strictly to the dietary guidelines. Additionally, they were informed about the child's prognosis and received genetic counseling, which included discussing the potential inheritance of the defect in future offspring.

## Discussion

Fructose bisphosphatase regulates gluconeogenesis by converting fructose-1,6-biphosphate into fructose-6-phosphate and inorganic phosphate. This condition is inherited in an autosomal recessive pattern and causes severe, recurrent episodes of life-threatening hypoglycemia and metabolic acidosis in children, often appearing in infancy, as seen in our case [8]. This genetic mutation leads to a lack of fructose-1,6-diphosphatase activity, impairing gluconeogenesis in the liver and causing the accumulation of gluconeogenic precursors from dietary intake, such as fructose, lactate, glycerol, alanine, and other amino acids [9].

It was found in another case study that a novel maternal mutation (c.977(exon7)T>C) in the *FBP1* gene was identified in two siblings: a two-year-old female and a newborn male. Clinical suspicion for FBP1 deficiency should be heightened in cases presenting with acute infection onset, severe metabolic acidosis, and hypoglycemia. Early genetic sequencing can confirm the diagnosis, facilitating timely intervention and sustained dietary management to prevent mortality, support growth, and improve the quality of life for affected children [10].

Due to the disease's nonspecific clinical features, differential diagnoses such as glycogen storage diseases (GSDs) and mitochondrial diseases (MDs) should be considered at first and ruled out before confirming enzymatic defects such as FBP1 deficiency, as these conditions also present with similar symptoms of hypoglycemia and hyperlactatemia [11]. In another case series, three cases of fructose intolerance were documented, with manifestations including hypoglycemia, recurrent metabolic acidosis, abdominal distension, and hepatomegaly. Genetic testing revealed mutations in the *FBP1* gene in two patients and one mutation in the *ALDO-B* gene [12].

Gluconeogenesis primarily occurs in the liver and kidneys, and some of these conditions lead to the accumulation of toxic metabolites, which can result in end-organ damage leading to hepatomegaly as one of the presentations [13,14]. In our case, the child presented with recurrent episodes of vomiting, hypoglycemia, and metabolic acidosis, without features of abdominal distension or hepatomegaly. While hypoglycemia episodes are uncommon in neonates due to lower glycogen stores, they typically occur more frequently in older infants [15]. In a case reported by Madhusudan et al., they described a 2.5-year-old boy with normal development who experienced recurrent hypoglycemic seizures. Laboratory analyses during critical episodes showed ketosis, lactic acidosis, hyperuricemia, and elevated triglycerides. Initially provisionally diagnosed with GSD type 1; subsequent evaluations did not reveal the typical physical features associated with this condition [1].

Historically, the definitive diagnosis of FBP1 deficiency relied on enzymatic activity assays conducted on liver biopsy samples or cultured leukocytes [16,17]. However, molecular analysis of the *FBP1* gene has now largely supplanted these methods. The *FBP1* gene is located on chromosome 9q22.2-q22.3 and encodes the enzyme (P09467; NM_000507; transcript id ENST00000375326.8), spanning seven coding exons over 31 kilobases, interspersed with six introns. Initial research on homozygous and heterozygous mutations in the *FBP1* gene was pioneered in the Japanese population, with subsequent studies identifying specific mutations common to various regions [7].

In studies conducted in India and Pakistan by Ijaz et al., a DNA nucleotide change (c.611_614delAAAA) resulting in a predicted protein change (p.Lys204ArgfsTer72) was observed [7]. Afroze et al. in 2013 identified another DNA nucleotide change (c.841G>A) leading to a predicted protein change (p.Glu281Lys), observed in populations from India, Pakistan, and Saudi Arabia [3]. These recent advancements in genetic testing are helping us identify various mutations across different countries resulting in the presentation of this condition across children of various age groups and ethnicities.

## Conclusions

FBP1 deficiency is a spectrum disorder that should be considered in children presenting with symptoms such as failure to thrive, recurrent hypoglycemia, and/or metabolic acidosis. These children may experience hypoglycemia when consuming fructose-rich diets. Differential diagnoses include other gluconeogenesis disorders such as GSD type 1, pyruvate carboxylase deficiency, and phosphoenolpyruvate carboxykinase deficiency, as well as mitochondrial disorders, all of which present with ketotic hypoglycemia and lactic acidosis. It is essential to exclude these conditions before diagnosing FBP1 deficiency, which usually does not cause end-organ damage.

This case study illustrates a comprehensive approach to diagnosing and managing a female child, aged one year and six months, with FBP1 deficiency, identified via clinical exome sequencing as a pathogenic homozygous variant in the *FBP1* gene. Her symptoms, including sudden-onset vomiting, hypoglycemia, and abnormal metabolic markers, were effectively managed with a specific dietary plan designed to prevent fasting and regulate carbohydrate intake. Ongoing follow-ups confirmed normalized metabolic parameters and growth. The treatment focuses on dietary management to prevent hypoglycemia, emphasizing the restriction of fructose and sorbitol and careful food intake monitoring.
